# Routine intubation in the prone position

**DOI:** 10.3109/03009734.2012.686125

**Published:** 2012-10-30

**Authors:** Klaus Baer, Bo Nyström

**Affiliations:** Clinic of Spinal Surgery, Löt, 64594 Strängnäs, Sweden

**Keywords:** Anaesthesia, endotracheal intubation, laryngoscopy, pharyngeal anatomy, prone position

## Abstract

**Background.:**

Tracheal intubation in the prone position has previously been reported only as a necessity in a very few emergency situations. It emerged at our clinic as a routine after invention of a test aimed at pinpointing a painful motion segment in patients with chronic low back pain who were candidates for lumbar fusion operation.

**Material and methods.:**

During a 6-year period 247 consecutive patients were treated at our clinic, 91 men and 156 women, mean age 42.8 years, range 25.3–62.8. Classification of the pharyngeal structures according to Mallampati et al. was done the day before surgery, and grading of visualization of the glottis as described by Cormack and Lehane was done during intubation, with the aim of revealing factors of importance for the possibility of performing tracheal intubation in the prone position.

**Results.:**

The large majority of patients classified preoperatively as Mallampati class 1 had Cormack and Lehane grade 1 at laryngoscopy, although some patients had grades 2, 3, and 4. Most problems with intubation in the prone position were anticipated among those classified preoperatively as Mallampati class 3, but tracheal intubation in the prone position was still possible in 21 of the 23 patients in this group. In all, tracheal intubation in the prone position was successful in 244 of the 247 patients (98.8%).

**Conclusion.:**

Routine tracheal intubation in the prone position can be performed effectively by experienced anaesthesiologists, but this requires continuous training and good support from the anaesthesiology staff.

## Introduction

Spine surgery is almost invariably performed with the patient in the prone position. Development of this position and its physiological effects and risks were recently reviewed by Edgcombe et al. (1). In this planned type of surgery tracheal intubation has been performed in the supine position and the patient then turned to the prone position for the surgical procedure. The need for tracheal intubation in the prone position has been reported in only a few emergency situations (2,3), and this need has been met (2) or resolved by using a laryngeal mask (4,5). In some types of surgery in the prone position with short operation times, induction of anaesthesia and use of a laryngeal mask with the patient in the prone position facilitate the procedure, since the patient can position himself/herself comfortably (6–8).

Spine surgery with long operation times such as in fusion surgery is a good example in which induction of anaesthesia and tracheal intubation are normally performed in the supine position, after which the patient is turned to the prone position and positioned on a framework to minimize intra-abdominal pressure and thereby bleeding, and also possible pressure on the knees, hips, male genitals, face, and eyes.

At our clinic a test aimed at pinpointing a painful motion segment in patients with chronic low back pain was invented in order to select as accurately as possible the segment that might be responsible for the patient's back pain. After performing this test under local anaesthesia with the patient awake in the prone position and an open surgical wound in the lumbar back, we found that turning the patient to the supine position for induction of anaesthesia and intubation was time-consuming and somewhat risky regarding infection. The present article describes our experience using routine intubation in the prone position under these circumstances.

## Material and methods

The study represents the experience of a single centre over a 6-year period in treating a group of 247 consecutive patients (91 men and 156 women, mean age 42.8 years, range 25.3–62.8), all subjected to prone intubation after giving their informed consent. All had ASA status I or II. Preoperatively the range of neck movement was tested, and the visibility of the pharyngeal structures according to Mallampati et al. (9) was documented. During intubation the following day the extent of exposure of the glottis was graded according to Cormack and Lehane (10) in order to examine and determine what observations were important for the final goal, i.e. the possibility of performing prone tracheal intubation.

### Open mechanical provocation test

With the patient awake and in the prone position, mepivacaine + adrenaline, 5 mg/mL + 5 μg/mL, usually 15–20 mL, are administered subcutaneously over the spinal processes in the lower lumbar region. The processes are exposed and gently tapped, permitting patients to report about recognition and localization of their ordinary pain. If there seems to be a definitive localization of the patient's pain to a specific segment, fusion of that segment is decided upon. Since the patients had to be focused and as mentally clear as possible concerning the localization of their back pain, no premedication was given.

### Anaesthesia and laryngeal intubation procedure

Prior to anaesthesia induction, laryngoscopes and tubes of different sizes, and stylets for use inside the tube if necessary, are at hand. The patient lies prone on the operating table with the head turned to the right. A nurse anaesthetist stands on the left side of the table to assist and support the patient's head during induction. The anaesthesiologist stands at the head of the table and informs the patient regarding the procedure. Fentanyl 0.2 mg i.v. is given as premedication. The anaesthesiologist places his/her right hand underneath the patient's head and elevates it slightly, and the mask is applied and oxygen 50% is administered. Preoxygenation continues into assisted spontaneous ventilation. Blood pressure (NIBP), ventilatory frequency, and oxygenation are checked continuously by pulse oxymetry. During assisted ventilation midazolam 0.2 mg/kg is given i.v. Oxygen saturation as determined by pulse oxymetry should be at least 98%. When the patient is asleep and well ventilated, rocuronium bromide 0.6 mg/kg is given i.v. for relaxation. When the patient is relaxed, verified by muscle stimulation, the head is elevated by the anaesthesiologist using the right index finger around the upper molars while at the same time using the left hand to elevate and slightly extend the head dorsally. The Macintosh laryngoscope with an adult blade is cautiously applied with the left hand until the right part of the tongue is pressed down and no longer visible. The epiglottis is localized, the laryngoscope adjusted, and the glottis inspected. Laryngeal intubation is performed with a smaller tube at hand as well as a stylet for insertion into the tube in case of problems in getting the tube in place. If intubation in the prone position turned out to be impossible the decision to discontinue the attempt was taken at 2 minutes. The patient was manually ventilated, a sterile dressing was applied to the open surgical wound in the patient's back, and the patient turned to the supine position for intubation. Staff training in this procedure is done regularly, and an extra operating table is always on standby in case this happens. The practical performance of tracheal intubation in the prone position has been described previously in more detail (11,12). When considered necessary to facilitate laryngeal intubation, external laryngeal manipulation was applied (BURP manoeuvre = backward, upward, and rightward pressure on the larynx) (13).

## Results

Of the 247 patients investigated and treated during the study period, 163 were classified preoperatively as Mallampati class 1. Among them there was one patient in whom not even the epiglottis could be seen at laryngoscopy, Cormack and Lehane grade 4; in this patient intubation in the prone position was not possible (Table I).

**Table I.** Correlation between Mallampati grading of pharyngeal structures (see text) seen before laryngoscopy and visualization of the glottis according to Cormack and Lehane at intubation in 247 consecutive patients undergoing spinal fusion surgery.

	Cormack and Lehane grade				
Mallampati class	G 1	G 2	G 3	G 4	Total
C 1	137	23	2	1^a^	163
C 2	22	26	13	0	61
C 3	3	8	8	2 + 2^a^	23
Total	162	57	23	5	247

^a^Patients in whom intubation in the prone position was not possible.

Sixty-one patients were preoperatively classified as Mallampati class 2, and all were possible to intubate in the prone position. In 8 of the 23 patients classified preoperatively as Mallampati class 3 only the epiglottis was seen, but intubation in the prone position was still possible. In another four not even the epiglottis could be seen at laryngoscopy. It was nevertheless possible to perform intubation in the prone position in two of them by using the BURP manoeuvre (Table I). Thus in 3 out of the 247 patients intubation in the prone position was not possible (1.2%).

Intubation was always completed within a maximum of 2 minutes following rocuronium injection. Extubation after surgery was usually performed in the prone position when the patient was awake and breathing spontaneously. No systematic difference was noted between men and women concerning difficulties during this intubation procedure. There were no complications from the procedure, although one patient sustained a tooth injury.

## Discussion

In ordinary clinical work there is seldom a need for tracheal intubation in the prone position, but in extreme emergency situations such a need may arise (2,3). In these situations fibre-optic intubation may be considered, but this technique requires the patient's co-operation, special equipment, and extensive training as noted by van Zundert et al. (2). These authors tried fibre-optic intubation without success in a patient with a traumatic thoracic injury, but handled the situation using direct laryngoscopy in the prone position.

Although it was found during this study that preoperative inspection of the patient's pharyngeal structures was of value in preparing for the intubation, there were exceptional patients with Mallampati class 1 who were classified at laryngoscopy as grades 3 and 4 according to Cormack and Lehane (10). Such a discrepancy between visibility of the pharyngeal structures and the findings at laryngoscopy was also noted by Charters et al. (14), who abandoned oral inspection as a useful tool for predicting difficult tracheal intubation.

In previous studies the sensitivity of the Mallampati test has been found to vary from 33 to 84, and the specificity from 65 to 89 (15–19). In the original study by Mallampati et al. (9) the figures were 50 and 99.5, respectively, and in our study they are 42.8 and 94.9, respectively. The reason for these differences is probably differences between samples of patients as well as differences in evaluations of the Mallampati and Cormack and Lehane scales. Differences are also reported concerning whether or not Mallampati class 2 should predict a difficult intubation (15). Although the test has a low sensitivity and specificity, there is no doubt that most problems with tracheal intubation occur in patients with less visualization of the pharyngeal structures, as seen in our study and also previously in larger studies with patients in the supine position (20,21).

Among the 247 patients in our study, failure to perform intubation in the prone position occurred in only 3 (1.2%), which is markedly higher than the incidence of failed tracheal intubations in the supine position (22) but nevertheless very low. The present study shows that laryngeal intubation in the prone position can be performed almost as well as in the supine position in young and middle-aged patients. However, continuous training and good support from the anaesthesiology staff are necessary.
